# Metabolic syndrome in rats is associated with erectile dysfunction by impairing PI3K/Akt/eNOS activity

**DOI:** 10.1038/s41598-017-12907-1

**Published:** 2017-10-18

**Authors:** Rui Li, Kai Cui, Kang Liu, Hao Li, Yan Zhang, Xiaming Liu, Ruibao Chen, Mingchao Li, Tao Wang, Shaogang Wang, Jihong Liu, Ke Rao

**Affiliations:** 1 Department of Urology, Tongji Hospital, Tongji Medical College, Huazhong University of Science and Technology, Wuhan, China; 2 Institute of Urology, Tongji Hospital, Tongji Medical College, Huazhong University of Science and Technology, Wuhan, China

## Abstract

Metabolic syndrome (MetS) is a risk factor for erectile dysfunction (ED), but the underlying mechanisms are unclear. The aims of this study were to determine the underlying mechanisms of metabolic syndrome-related ED (MED). Sprague Dawley (SD) rats were fed a high-fat diet for 6 months, and metabolic parameters were then assessed. An apomorphine test was conducted to confirm MED. Only rats with MED were administered an intracavernosal injection of either epidermal growth factor (EGF) or vehicle for 4 weeks. Erectile responses were evaluated by determining the mean arterial blood pressure (MAP) and intracavernosal pressure (ICP). Levels of protein expression were examined by western blotting and immunohistochemistry. Body weight, fasting blood glucose, plasma insulin and plasma total cholesterol were increased in the MetS rats compared with those in control rats (each﻿ p < 0.05). The  maximum ICP/MAP, total ICP/MAP and concentration of cyclic guanosine mono-phosphate (cGMP) were significantly decreased in MED rats (each p < 0.05). The expression levels of p110α, p-Akt1 (Tyr308)/Akt1 and p-eNOS (Ser1177)/eNOS were reduced in MED rats (each p < 0.05). Activation of the PI3K/Akt/eNOS signaling cascade (intracavernosal injection of EGF) reversed these changes (each p < 0.05). The present study demonstrates that downregulation of the PI3K/Akt/eNOS signaling pathway is involved in MED.

## Introduction

Erectile dysfunction (ED), which is the inability to achieve and sustain an erection sufficient for satisfactory vaginal intercourse, is a common clinical condition that affects quality of life^1^. ED affects 5–20% of men worldwide, and metabolic syndrome (MetS) has been proven to be a risk factor^2–4^. MetS, a disease arising from the worldwide obesity epidemic, manifests as insulin resistance, hypertension, hyperlipidemia, and diabetes. The definition of MetS varies somewhat in the literature, but the presence of three or more of the aforementioned components is typically defined as MetS^5^. MetS has a high prevalence around the world and has been reported to be as high as 9.5% in Europe and 35–39% in the US^6^. Furthermore, the prevalence of ED in MetS patients has been reported to be twofold greater than in control patients^7^. Phosphodiesterase type 5 inhibitor (PDE5i) therapy, which is currently the first-line treatment for ED, remains markedly inefficient in this population, and therefore, new therapeutic methods are needed^8^. Hence, the aims of this study were to determine the underlying mechanisms of metabolic syndrome-related ED (MED).

The phosphatidylinositol 3-kinase (PI3K)/protein kinase B (PKB/Akt) signaling pathway is one of the main regulatory networks in the cell and influences almost all cellular activities, including replication, growth, metabolism, movement and differentiation. PI3K comprises three classes of enzymes (classes I–III). Class IA PI3K is a heterodimeric kinase that consists of a p110 catalytic subunit and a p85 regulatory subunit and plays an important role in human cell biology. The class IA p110 isoform is a complex composed of p110α, p110β and p110γ, while p85 isoforms contain p85α and p85β^9^. PI3K is activated by various extracellular signals, such as epidermal growth factor receptor (EGFR) or insulin-like growth factor1receptor (IGF1R). Akt is a downstream serine/threonine protein kinase that acts as a core signaling input to regulate downstream effectors and preserve cell homeostasis. Akt comprises three highly conserved homologous isoforms: Akt1 (PKBα), Akt2 (PKBβ), and Akt3 (PKBγ)^10^. Three Akt isoforms all contain a C-terminal hydrophobic domain and a N-terminal regulatory pleckstrin homology (PH) domain, a central kinase domain with serine/threonine specificity^11,12^. Studies have shown that Akt isoforms are similar in structure but distinct in function in physiological and pathological processes, partly due to different tissue-specific expression of Akt isoforms. For example, although Akt1 expression is ubiquitous, whereas Akt2 is highly expressed in adipocytes and muscle and plays an important role in glucose homeostasis. The distribution of Akt3 is more restricted and is primarily expressed in the testes and brain^13–16^. Upon activation, Akt phosphorylates many downstream substrates, which regulate protein synthesis and cell growth. Activated Akt can directly activate endothelial nitric oxide synthase (eNOS) via phosphorylation at Ser1177, leading to augmented nitric oxide (NO) synthesis^17^. Previous work revealed that the Akt-dependent phosphorylation of eNOS mediates penile erection^18^. Other studies revealed that Akt-dependent phosphorylation and activation of eNOS leads to sustained NO production and maximal erection^19,20^. Similarly, Wen *et al*. demonstrated that the A_2B_ adenosine receptor contributes to penile erection via PI3K/Akt signaling-mediated eNOS activation^21^. However, the role of the PI3K/Akt/eNOS signaling pathway in MED remains to be elucidated.

The aims of the present study were to determine the effect of the PI3K/Akt/eNOS signaling pathway on erectile function in a rat *in vivo* model of MED and to suggest a potential novel treatment strategy for MED.

## Results

### Metabolic Parameters

Body weight, fasting blood glucose, plasma total cholesterol and insulin levels in rats fed the high-fat diet for 6 months were significantly higher than those in the age-matched controls. No significant differences in blood pressure, triglycerides, low-density-lipoprotein (LDL) and high-density-lipoprotein (HDL) were found among the groups (Table 1).

**Table 1:** Effect of the high-fat diet on general parameters.

	CO	MED	EGF
Body weight (g)	577.0 ± 27.0	640.0 ± 39.0*	634.0 ± 48.0*
Fasting blood glucose (mmol/L)	6.4 ± 0.5	7.6 ± 0.8*	7.5 ± 1.1*
Blood pressure (mmHg)	121 ± 18	128 ± 15	124 ± 17
Insulin level (mIU/L)	16.0 ± 1.1	20.6 ± 1.6*	20.5 ± 1.7*
Total cholesterol (mmol/L)	1.95 ± 0.08	4.32 ± 1.05*	2.68 ± 0.26^#^
Triglycerides (mmol/L)	0.64 ± 0.08	0.59 ± 0.10	0.68 ± 0.07
High density lipoprotein (mmol/L)	2.81 ± 0.61	3.11 ± 1.15	3.35 ± 1.47
Low density lipoprotein (mmol/L)	2.27 ± 0.12	2.35 ± 0.16	2.21 ± 0.13

Data are shown as the means ± SD of 5~7 rats. *p < 0.05 compared with the CO group, ^#^p < 0.05 compared with the MED group. CO, normal control rats; MED, metabolic syndrome-related erectile dysfunction rats; EGF, MED rats treated with epithelial growth factor; SD, standard deviation.

### Upregulation of the PI3K/Akt/eNOS Signaling Pathway Improves Erectile Function in MED Rats

**Figure 1:**
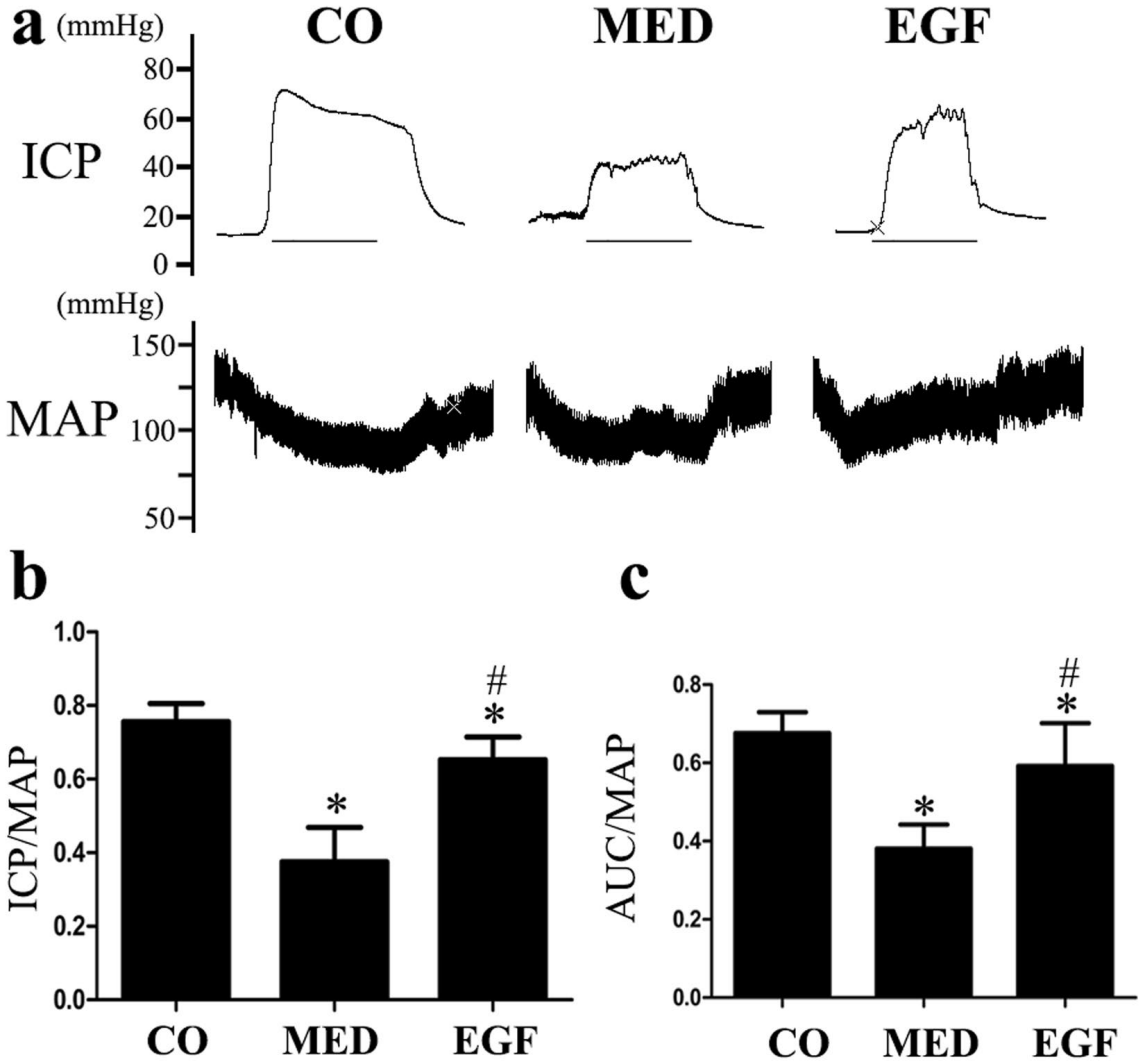
(**a**) Evaluation of erectile function via electrical stimulation of the cavernous nerve. Representative carotid artery pressure and intracavernous pressure tracings obtained following cavernous nerve stimulation at 5 V for 1 min, respectively, in the CO, MED and EGF rats. (**b,c**) Erectile function presented as the maximum ICP/MAP and AUC/MAP in the three groups. Data are expressed as the means ± SD of 5~7 rats. *p < 0.05 vs the CO group, ^#^p < 0.05 vs the MED group. CO, normal control rats; MED, metabolic syndrome-related erectile dysfunction rats; EGF, MED rats treated with epithelial growth factor; ICP, intracavernosal pressure; MAP, mean arterial pressure; AUC, total intracavernosal pressure; SD, standard deviation.

The effect of the PI3K/Akt/eNOS signaling pathway upregulation on the recovery of erectile function is illustrated in Fig. 1. The control group exhibited normal maximum (max) intracavernous pressure (ICP)/mean arterial blood pressure (MAP) and total ICP/MAP, whereas MetS consistently caused ED. The max ICP/MAP and total ICP/MAP ratios were lower in the MED group than in the control group. Partial but significant recovery of erectile function was observed in the epidermal growth factor (EGF)-treated groups compared with the MED group, as reflected by significantly higher max ICP/MAP and total ICP/MAP ratios in response to cavernous nerve electrical stimulation (p < 0.05).

### EGF Treatment Upregulates EGF Expression in MED Rats

**Figure 2:**
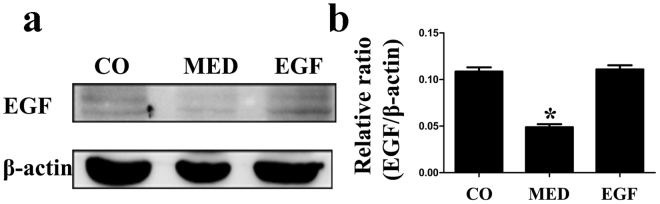
(**a,b**) Western blot analysis of epidermal growth factor (EGF) expression normalized to β-actin levels in the CO, MED and EGF groups. Data in the bar graphs are expressed as the means ± SD of 5~7 rats. *p < 0.05 vs the CO group. CO, normal control rats; MED, metabolic syndrome-related erectile dysfunction rats; EGF, MED rats treated with epithelial growth factor; SD, standard deviation.

To clarify the mechanism of PI3K/Akt/eNOS activation, we examined EGF expression by western blotting. In the penis, EGF protein expression levels were significantly reduced in MED rats. EGF administration completely reversed the decrease. Statistically significant differences in EGF expression were observed in the three groups (Fig 2a,b).

### EGF Treatment Upregulates PI3K Expression in MED Rats

**Figure 3:**
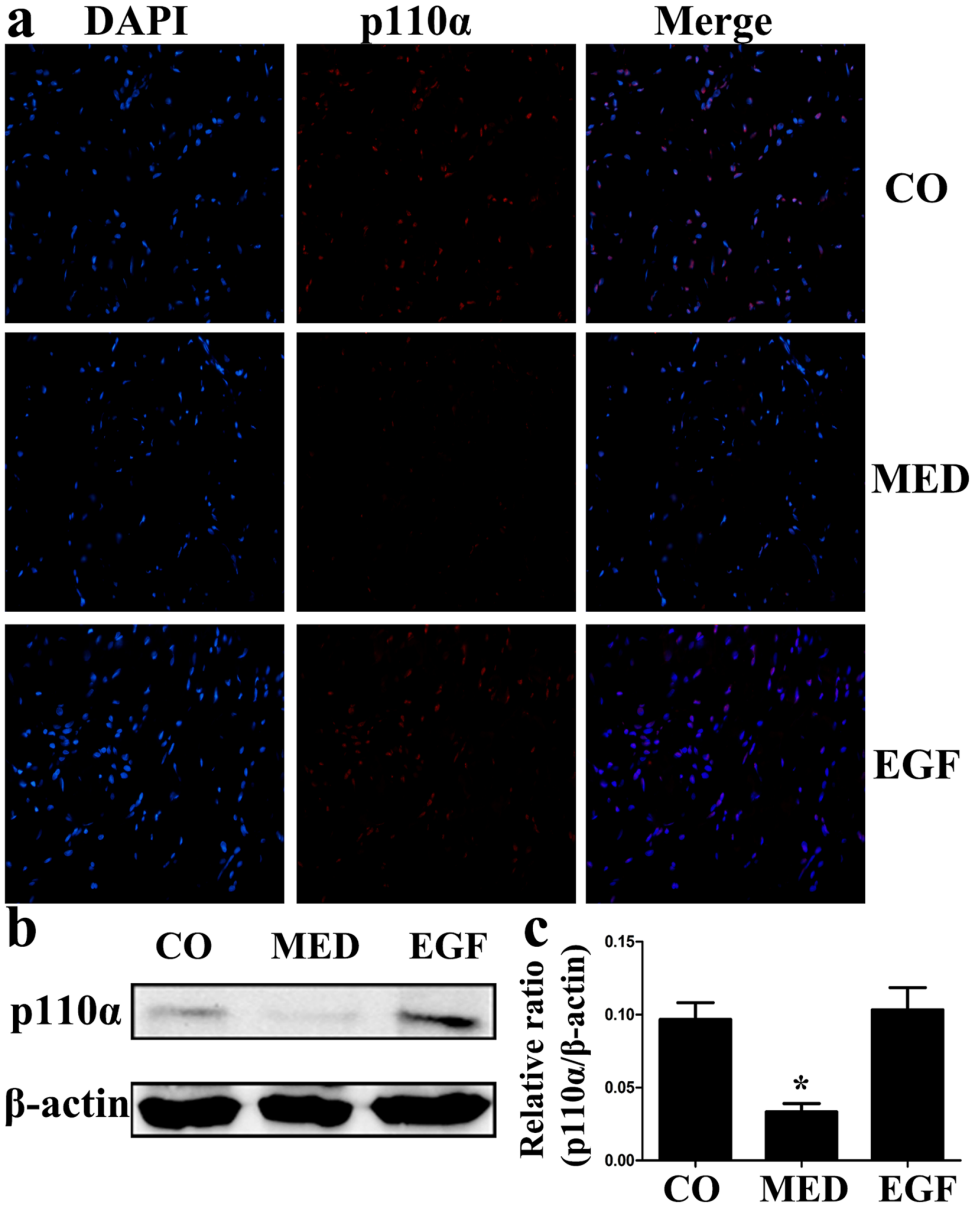
(**a**) Immunofluorescence staining of cavernous tissue using an antibody against p110α in the CO, MED and EGF groups. (**b,c**) Western blot analysis of p110α expression normalized to β-actin levels in the three groups. Data in the bar graphs are expressed as the means ± SD of 5~7 rats. *p < 0.05 vs the CO group. CO, normal control rats; MED, metabolic syndrome-related erectile dysfunction rats; EGF, MED rats treated with epithelial growth factor; SD, standard deviation.

The expression levels of PI3K subunits were determined in all three groups. Baseline p110α levels were reduced in the MED group compared with those in the control group, and EGF treatment increased p110α protein expression (Fig. 3b,c). The expression levels of other PI3K subunits were similar in all three groups. We also performed immunofluorescence staining for p110α. Only faint staining was visible in the MED group, indicating little p110α protein expression in the MED rats. In EGF-treated rats, p110α staining revealed strong expression (as assessed by five high-powered fields per slide; Fig. 3a).

### EGF Treatment Upregulates Akt1 Phosphorylation in MED Rats

**Figure 4:**
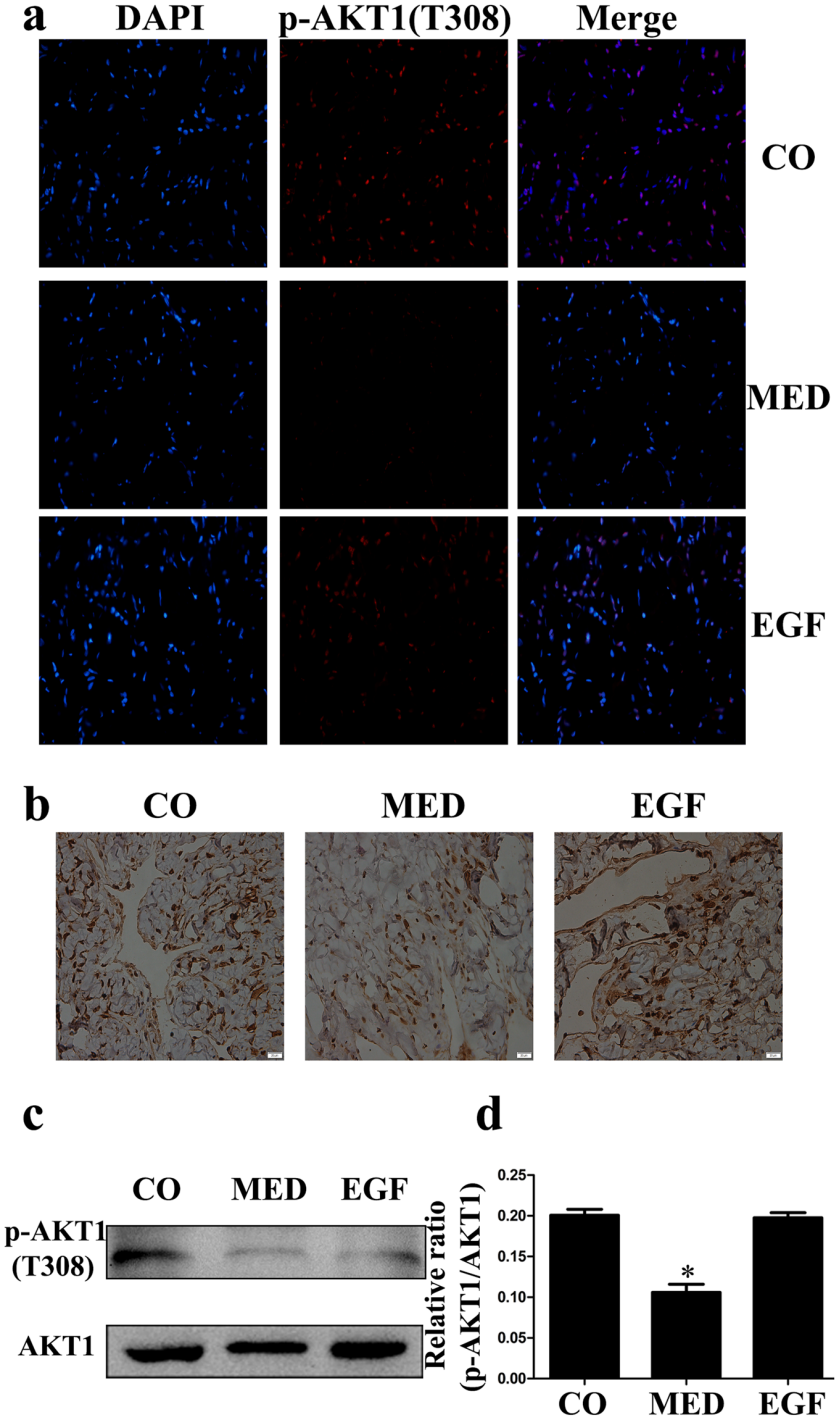
(**a**) Immunofluorescence staining of cavernous tissue using an antibody against p-Akt1(Tyr308) in the CO, MED and EGF groups. (**b**) Immunohistochemistry staining of cavernous tissue was performed with an antibody against Akt1 in three groups (magnification: ×400 scale bar: 20 μm). (**c,d**) Western blot analysis of p-Akt1 (Tyr308) and Akt1 expression in the three groups. Data in the bar graphs are expressed as the means ± SD of 5~7 rats. *p < 0.05 vs the CO group. p-Akt, phosphor-protein kinase B; CO, normal control rats; MED, metabolic syndrome-related erectile dysfunction rats; EGF, MED rats treated with epithelial growth factor; SD, standard deviation.

We performed immunohistochemical staining and western blotting of cavernous tissue with antibodies against Akt1 and p-Akt1 (Tyr 308) in all three groups. Representative photomicrographs of each group are shown in Fig. 4. We detected significantly lower Akt1 (Tyr 308) phosphorylation in the MED rats than in the control rats. After intracavernous administration of EGF, Akt1 (Tyr308) phosphorylation showed a clear increase compared with that in the MED group, as determined by immunohistochemical and western blot analyses (Fig. 4a,b,c).

### EGF Treatment Upregulates eNOS Phosphorylation in MED Rats

**Figure 5:**
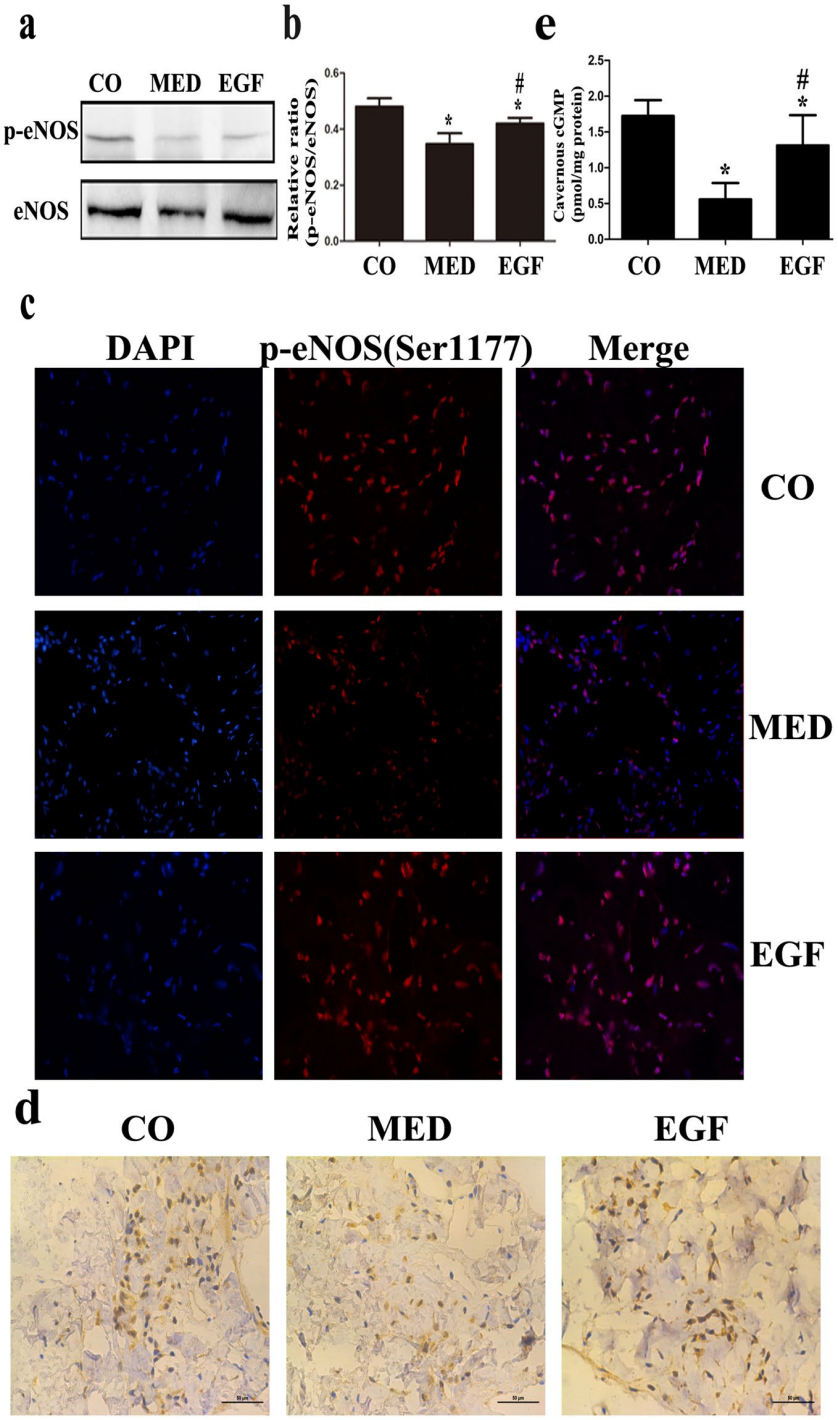
(**a,b**) Western blot analysis of eNOS and p-eNOS (Ser1177) expression in the CO, MED and EGF groups. (**c**) Immunofluorescence staining of cavernous tissue using an antibody against p-eNOS (Ser1177) in the three groups. (magnification: ×400). (**d**) Immunohistochemistry staining of cavernous tissue was performed with an antibody against eNOS in three groups. (magnification: × 400). (**e**) cGMP concentrations in the three groups. Data in the bar graphs are expressed as the means ± SD of 5~7 rats. *p < 0.05 vs the CO group, ^#^p < 0.05 vs the MED group. eNOS, endothelial nitric oxide synthase; CO, normal control rats; MED, metabolic syndrome-related erectile dysfunction rats; EGF, MED rats treated with epithelial growth factor; SD, standard deviation.

We next evaluated the expression of phospho-eNOS (p-eNOS, Ser1177) and eNOS in the corpus cavernosum. In MED rats, the basal p-eNOS (Ser1177)/eNOS ratio was significantly reduced compared with that in the age-matched controls. At 4 weeks after intracavernous EGF treatment, eNOS phosphorylation increased compared with that in the MED rats, as determined by western blotting and immunohistochemistry (Fig. 5a–d). We also measured cavernous tissue cyclic guanosine mono-phosphate (cGMP) concentrations in all three groups. Cavernous cGMP levels decreased significantly in MED rats compared with those in controls. Cavernous cGMP concentrations were markedly increased in MED rats treated with EGF but were still lower than those in the controls (Fig. 5e).

## Discussion

In this study, we demonstrated that EGF contributes to penile erection via eNOS activation in a PI3K/Akt-dependent manner in MED rats. Significantly reduced erectile function was observed following downregulation of the PI3K/Akt/eNOS signaling pathway in MED rats. Mechanistically, we demonstrated that upregulation of the PI3K/Akt/eNOS signaling pathway is a dual mechanism that ameliorates erectile function in MED rats. Thus, our study reveals a previously unrecognized role for PI3K/Akt/eNOS signaling pathway in penile erection in MED rats and offers a potential novel therapeutic strategy for MED rats.

ED is known to be more common in men with obesity, type 2 diabetes mellitus, and/or MetS^22^. Due to the popularity of western diets, an increasing number of people suffer from obesity, high blood pressure, diabetes, insulin resistance, high blood lipids and/or MetS, and subsequently, the prevalence of ED caused by the above symptoms is also increasing. Although MetS animals are commercially available, these transgenic mice are not representative of the natural course of MetS. To mimic the natural processes of MED in the general population, an MED model was established by feeding rats a high-fat diet. A recent study using an animal model of type 1 diabetes-associated ED revealed that impairment of the PI3K/Akt/eNOS signaling pathway in the penis is associated with reduced erectile capability^23^. Another study reported that metabolic disorders lead to abnormal endothelial function that reduces eNOS generation and decreases eNOS phosphorylation at Ser1177 and that activation of the PI3K/Akt/eNOS signaling pathway reverses endothelial dysfunction^18^. Hence, the PI3K/Akt/eNOS signaling pathway may be the mechanism responsible for MED. In the current study, we established an MED model and found that erectile function in MED rats was lower than that in control rats. We then investigated the molecular mechanisms and found downregulation of the PI3K/Akt/eNOS signaling pathway, decreased expression of PI3K subunit p110α, decreased levels of phosphorylated Akt1 and eNOS, and decreased corpus cavernosum cGMP concentrations in MED rats. These data demonstrate that downregulation of the PI3K/Akt/eNOS signaling pathway is involved in MED. Therefore, the PI3K/Akt/eNOS signaling pathway might be an appropriate therapeutic option for MED.

EGF is a member of the growth factor family with many biological effects, including regulating cell replication, cell movement and cell survival. PI3K transduces signals from various cytokines and growth factors into intracellular messages by generating phospholipids, which activate downstream effectors including Akt and eNOS. The PI3K/Akt pathway regulates the phosphorylation of eNOS at Ser1177, which causes an increase in NO, leading to the accumulation of cGMP^24–26^. Studies have reported that insulin-like growth factor-1 (IGF-1) improves erectile function in diabetic and aging rats through increased eNOS expression^27,28^. However, how elevated eNOS is induced by IGF-1 remains unclear. In the current study, we used an intracavernous injection of EGF to investigate erectile responses. According to our ICP data, erectile function markedly recovered after EGF treatment, indicating a therapeutic effect. We found that the recovery of erectile function in MED rats by EGF administration was triggered at the molecular level by activation of the PI3K/Akt/eNOS signaling pathway. eNOS activation caused the sustained production of NO, which promotes cavernosal smooth muscle relaxation and the maintenance of an erection. In our study, we demonstrated that p110α and phosphorylated Akt1 (Tyr308) and eNOS (Ser1177) were reduced in MED rats. Treatment with EGF, which actives the PI3K/Akt/eNOS signaling pathway, increased the expression of p110α and the phosphorylation of Akt1 (Tyr308) and eNOS (Ser1177). This finding strongly suggests that EGF activates the PI3K/Akt pathway, promoting increased phosphorylation and, thus, activation of eNOS. The latter increases NO availability and produces larger quantities of cGMP, which maintain penile erection.

However, our work has some limitations. First, we did not investigate how EGF regulates the PI3K/Akt/eNOS signaling pathway. Second, the recovery of erectile function was relatively short-lived. Further studies are needed to test whether repeated intracavernous injections of EGF can induce more durable recovery of erectile function.

In summary, the PI3K/Akt/eNOS signaling pathway is involved in MED. We offer strong evidence demonstrating that EGF successfully actives the PI3K/Akt/eNOS signaling pathway, leading to an increase in the cGMP concentration and improvement of erectile function in MED rats. Therefore, our study offers novel findings and thereby insights into the molecular mechanisms of MED. Further studies that elucidate the precise mechanisms are warranted.

## Materials and Methods

### Experimental Outline

All animal studies were approved by the Committees on Animal Experiments at Tongji Hospital (Tongji Medical College, Huazhong University of Science and Technology, Wuhan, Hubei, China; IRB ID:TJ-A20150701), and all procedures complied with the Chinese Council on Animal Care Regulations for the care and use of laboratory animals.

A total of 70 male Sprague Dawley rats (3 weeks old) were randomly assigned to a control group (n=8) or the MetS group (n=62). The control group was fed a regular diet, and the MetS group was fed a high-fat diet for 6 months (Table 2). After 6 months, body weight, blood pressure, plasma insulin, fasting blood glucose and plasma lipid were assessed. MetS rats were chosen based on the results. Then, an apomorphine (APO) test was conducted to confirm MED^29–32^. The rats﻿ were habituated in a hanging cage for 10 min and received subcutaneous injection of APO in saline (80 mg/kg) into the loose skin of the neck. The erectile response of the tested rats was observed for 30 min. Rats that did not present an erectile response were considered MED rats. Only rats with MED were administered a signal intracavernosal injection of either EGF (1 µg/kg, Prospec, Ness Ziona, Israel) or vehicle for 4 weeks. Age-matched rats were given saline for 4 weeks. A tourniquet was applied at the base of the penis, and the needle was left in place for 3 min to allow the medication to diffuse throughout the cavernous space during intracavernosal injection.

**Table 2:** Diet Composition.

Control Diet		High-fat Diet		
Ingredients	%	Ingredients	gm	kcal
Corn	40	Casein, 80 Mesh	200	800
Soybean Meal	20	L-Cystine	3	12
Fish Meal	7	Corn Starch	0	0
Four	22	Maltodextrin 10	125	500
Yeast	4	Sucrose	68.8	275.2
Grease	1.5	Cellulose, BW200	50	0
Calcium Hydrogen Phosphate	2	Soybean Oil	25	225
Stone Powder	1.5	Lard	245	2205
Mineral Salts	0.7	Mineral Mix, S10026	10	0
Vitamins	0.3	DiCalcium Phosphate	13	0
Amino Acids	0.8	Calcium Carbonate	5.5	0
Choline	0.2	Potassium Citrate, 1 H_2_O	16.5	0
**Nutrient Content**	**%**	Vitamin Mix, V10001	10	40
Crude Protein	20.7	Choline Bitartrate	2	0
Crude Fiber	2.31	FD&C Blue Dye #1	0.05	0
Crude Fat	4.15	**Nutrient content**	**gm%**	**kcal%**
Calcium	1.24	Protein	26.2	20
Phosphorus	0.83	Carbohydrate	26.3	20
		Fat	34.9	60

### Measurement of Metabolic Parameters

After 6 months, blood was collected through the tail vein to determine plasma insulin and plasma lipid levels. Whole blood was centrifuged at 1580 g for 15 min at 4 °C. Plasma lipid and insulin levels were detected using a kit (Nanjing Jiancheng Bioengineering Institute, Nanjing, Jiangsu, China) according to the manufacturer’s instructions. Blood pressure was measured using a photoelectric tail-cuff system (AD Instruments PowerLab, Bella Vista, NSW, Australia) as described previously^33^, and fasting blood glucose was measured by obtaining a blood sample from the tail vein using a blood glucose meter (Johnson & Johnson, New Brunswick, NJ, USA).

### Measurement of Erectile Function

After 4 weeks of EGF treatment, erectile function was assessed in all rats. The assessments were performed as described previously^34^. First, the major pelvic ganglion and cavernous nerves were exposed and mounted onto stainless steel bipolar wire electrodes, which were connected to an electrical stimulator. The electrical stimulation parameters were as follows: electrical field stimulation at 5 V, a frequency of 15 Hz, a pulse width of 1.2 ms, and a duration of 1 min. Then, a PE-50 cannula (Becton Dickinson & Co., Sparks, MD, USA) was inserted into the carotid artery to monitor systemic MAP. Finally, a 25-gauge needle was inserted into one side of the corpus cavernosum to monitor ICP, connected to PE-50 tubing, and filled with 250 U/mL of a heparin solution. Both blood pressure and ICP were measured continuously using a data acquisition system (AD Instruments PowerLab/4SP, Bella Vista, NSW, Australia). The max ICP/MAP and total ICP/MAP were recorded for each rat. The animals were sacrificed via injection of 20 mL air, and the corporeal tissue was immediately collected from each rat for subsequent studies.

### Western Blotting

The assessments were performed as described by Li^34^. Lysates containing 40 mg protein were separated via SDS-PAGE and transferred to polyvinylidene fluoride membranes (Millipore Corp, Bedford, MA, USA). The primary antibodies used were anti-EGF (1:1000, Affinity, Zhenjiang, Jiangsu, China), anti-p110α (1:1000, Cell Signal Technology, Beverly, MA, USA), anti-Akt1 (1:500, Boster, Wuhan, Hubei, China), anti-phospho-Akt1 (Tyr308, 1:1000, Abcam, Cambridge, MA, USA), eNOS (1:1000, Abcam, Cambridge, MA, USA), p-eNOS (1:1000, Abcam, Cambridge, MA, USA) and β-actin (1:500, Multisciences, Hangzhou, Zhejiang, China). After hybridization with secondary antibodies, the samples were analyzed using a chemiluminescence detection system (Pierce, Thermo Fisher Scientific, Rockford, IL, USA).

### Immunohistochemistry and Immunofluorescence

Freshly dissected tissue was fixed in cold 4% formaldehyde and immersed overnight in buffer containing 30% sucrose. Tissues were then paraffin embedded until use. Sections were cut at 4 µm, adhered to charged slides, dewaxed with xylene, hydrated with an ethanol gradient, and treated with hydrogen peroxide to quench endogenous peroxidase activity (not required for immunofluorescence). After being rinsed, sections were washed three times in PBS for 5 min, followed by antigen repair. After excess fluid was drained, sections were incubated overnight at 4 °C with anti-p110α (1:50, Cell Signal Technology, Beverly, MA, USA), anti-Akt1 (1:50, Boster, Wuhan, Hubei, China), anti-phospho-Akt1 (Tyr308, 1:50, Abcam, Cambridge, MA, USA), eNOS (1:100, Abcam, Cambridge, MA, USA), or p-eNOS (1:100, Abcam, Cambridge, MA, USA). After several washes with PBS, the sections were incubated with Cy3-conjugated rabbit anti-IgG or Cy3-conjugated mouse anti-IgG for 60 min at room temperature. Finally, antigen-antibody reactions were developed using diaminobenzidine (DAB). For immunofluorescence studies, the sections were incubated with Cy3-conjugated rabbit anti-IgG or Cy3-conjugated mouse anti-IgG for 60 min at room temperature and then counterstained with DAPI (Beyotime Institute of Biotechnology, Haimen, Jiangsu, China). Signals were visualized on a microscope, and image analysis was performed using computerized densitometry in Image-Pro Plus (Media Cybernetics Inc., Bethesda, MD, USA).

### Measurement of Cavernous Tissue cGMP Levels

Corpus cavernosum cGMP concentrations were detected using an ELISA kit (Nanjing Jiancheng Bioengineering Institute, Nanjing, Jiangsu, China) according to the manufacturer’s instructions. The assays were conducted in duplicate, and the protein concentrations were measured to normalize the data.

### Statistical Analysis

The results were analyzed using SPSS 22.0 software (SPSS, Inc., Chicago, IL, USA) and are expressed as the means ± standard deviation (SD). All statistical analyses were performed using one-way analysis of variance (ANOVA) followed by Bonferroni’s multiple-comparison post-test to determine statistical significance (p < 0.05).
